# Exploring views on medical care for people with intellectual disabilities: an international concept mapping study

**DOI:** 10.1186/s12939-022-01700-w

**Published:** 2022-07-19

**Authors:** Marian E. J. Breuer, Esther J. Bakker-van Gijssel, Kristel Vlot-van Anrooij, Hilde Tobi, Geraline L. Leusink, Jenneken Naaldenberg

**Affiliations:** 1 Department of Primary and Community Care, Radboud University Medical Center, Radboud Institute for Health Sciences, Geert Grooteplein Noord 21, 6500 HB Nijmegen, The Netherlands; 2 Biometrics, Wageningen University & Research, Wageningen, The Netherlands

**Keywords:** ‘persons with intellectual disabilities’, ‘delivery of health care, Integrated’, ‘health services for persons with disabilities’, ‘medical care’, ‘concept mapping’, ‘inequities’

## Abstract

**Background:**

Medical care for people with intellectual and developmental disabilities (IDD) is organized differently across the globe and interpretation of the concept of medical care for people with IDD may vary across countries. Existing models of medical care are not tailored to the specific medical care needs of people with IDD. This study aims to provide an improved understanding of which aspects constitute medical care for people with IDD by exploring how international researchers and practitioners describe this care, using concept mapping.

**Methods:**

Twenty-five experts (researchers and practitioners) on medical care for people with IDD from 17 countries submitted statements on medical care in their country in a brainstorming session, using an online concept mapping tool. Next, they sorted all collected statements and rated them on importance.

**Results:**

Participants generated statements that reflect current medical and health care practice, their ideas on good practice, and aspirations for future medical and health care for people with IDD. Based on the sorting of all statements, a concept map was formed, covering 13 aspects that characterize medical and health care for people with IDD across nations. The 13 aspects varied minimally in importance ratings and were grouped into five overarching conceptual themes: (i) active patient role, (ii) provider role, (iii) context of care, (iv) consequences of care for people with IDD, and (v) quality of care.

**Conclusions:**

The themes, clusters and statements identified through this explorative study provide additional content and context for the specific patient group of people with IDD to the dimensions of previous models of medical care.

**Supplementary Information:**

The online version contains supplementary material available at 10.1186/s12939-022-01700-w.

## Background

People with intellectual and developmental disabilities (IDD) experience health inequities, and poorer health outcomes compared to the general population [1–3]. Some of these inequities can be prevented by improving medical care. For example, medical care professionals find it difficult to treat patients with IDD because of atypical presentations of complaints, different morbidity patterns, and more comorbidity and syndrome-related disorders compared with the general population [4–7]. Furthermore, communication and effective information exchange between medical care professionals and others involved is often problematic [8].

Countries have developed diverse approaches to provide medical care for people with IDD, ranging from enhancing mainstream medical care to developing specialized services [9–12]. Consequently, the organization of medical care differs internationally [9, 13–15] which makes comparison of the organization of medical care for people with IDD difficult. However, the diversity in approaches provides the opportunity to identify good practices and facilitate international understanding and learning.

Investigating differences in the international organization of medical care for people with IDD is difficult because there seems to be no generally accepted definition of medical care in literature [16, 17]. What we consider medical care may depend on country-specific cultural interpretations and institutional legacies [13]. Furthermore, the concepts medical care and health care are often used interchangeably. Overall, it is generally understood that medical care is a subset of health care. Medical care focuses on the diagnosis and treatment of an illness or injury of a person who needs medical attention, while health care focuses more broadly on promoting, maintaining, restoring, and monitoring the health of the public.

Existing models or frameworks of medical care are often focused on a specific part of this care, for example measuring access to medical care or quality of medical care, rather than describing medical care as a whole [17, 18]. More importantly, these models do not take into account aspects relating to specific medical care needs of people with IDD. Therefore, this study aims to provide an improved understanding of aspects of medical care for people with IDD by exploring how researchers and professionals describe medical care for people with ID through a concept mapping (CM) study.

## Methods

### Study design and procedures

CM, a mixed-methods participatory approach, was used [19]. The study consisted of nine steps, which will be explained in detail in the Data Collection and Analysis section and are summarized in Table 1. Steps 1, 2, 4, 5, 6, and 7 describe the common procedures for a CM study [18]. Groupwisdom™ software for CM (The Concept Systems® groupwisdom™ (Build 2019.24.01) [Web-based Platform], 2020) was used for data collection and analysis steps. In addition to the traditional CM procedure, the researchers performed three additional analyses beyond the scope of the used software to provide more insight in the data. Firstly, a qualitative analysis of the types of raw statements (step 3) was performed to better understand and represent the collected data, because the common CM procedure does not analyze the raw statements and only focuses on the content of the statements. Secondly, an additional sensitivity analysis (step 8) was performed to investigate the robustness of the final concept map. Lastly, a qualitative interpretation of the final cluster map (step 9) was performed to look for underlying dimensions within the data beyond the scope of the used software.

**Table 1:** Phases, activities, and time schedule of data collection and analysis

Phase	Activities	Result
1. Preparation brainstorming phase	Develop and pilot focus prompts (M.B., J.N., E.B., K.V.A., G.L.) •Advice Skype sessions with 3 researchers with expertise on medical care for people with intellectual and developmental disabilities (IDD) •Pre-pilot among 3 IDD physicians concerning focus prompt •Pilot study among 3 members of the IASSIDD Health SIRG Invite possible participants for the study (M.B., J.N., E.B., K.V.A., G.L.) •Create participant sampling plan •Create & send email invitation Make software ready to use (M.B., K.V.A.)	1 focus prompt
2. Brainstorming	25 participants create responses related to the focus prompt	92 raw statements
3. Qualitative analysis of raw statements	Qualitative analysis of 92 raw statements that were generated from the 25 participants, according to the following steps (M.B., J.N., H.T.): •Researchers individually analyze the 92 responses looking for underlying dimensions •Researchers compare their analyses and collaboratively decide on the underlying dimensions	92 raw statements with underlying dimensions
4. Preparation organization phase	Statement synthesis using the following procedure (M.B., J.N., E.B., G.L.): •Split up statements containing > 1 statement per sentence •Assign keywords to statements •Organize ideas based on keywords to bring overlapping statements together •Remove duplicates •Combine overlapping statements •Edit statements for clarity	92 raw statements reduced to a set of 79 unique statements
5. Organization	21 participants sort statements into piles of conceptually similar statements 18 participants rate statements on a 7-point Likert scale representing importance	79 statements individually sorted and rated
6. Preparation analysis phase	Assessment of sorting and rating data using the following criteria (M.B., J.N., E.B.): •Number of sorted statements •Number of created piles •Number of labelled piles (participants could create piles without labelling them) •Consistency of statements within piles •Time spent on the sorting and rating •Variation in rating (e.g., a participant’s rating data was excluded if the same rating was provided to all statements)	Sorting data of 3 participants excluded from analysis
7. Concept mapping analysis	Analysis using the following methods (M.B., J.N., E.B., K.V.A., H.T., G.L.): *A) Multidimensional scaling*: create a point map based on the sorting data, visualizing the relationship and proximity of statements to one another *B) Hierarchical cluster analysis*: create a cluster map by grouping statements that are closest to one another: •Research team collaboratively decides on upper and lower limits of the number of clusters •Researchers individually review the list of statements that are merged when moving from the highest desired number of clusters to the lowest, by looking at the average bridging values of the clusters and statements and the conceptual consistency of statements within clusters •Researchers individually decide the cluster size that retains most useful detail (further merging leads to non-interpretable cluster map) •Researchers collaboratively choose final cluster size and names by examining cluster statements *C) Analyze importance ratings*: Calculate mean importance rate for statements and clusters	A) Point map B) Cluster map C) Ratings of statements and clusters
8. Additional sensitivity analysis	*Jackknife resampling method*: Estimate sensitivity of the concept map by comparing the original allocation of statements within clusters with the 18 distributions resulting from systematically omitting one participant from the sample	Sensitivity for sampling variation
9. Qualitative analysis of final cluster map	Qualitative interpretation on the final cluster map	5 themes

### Participants

Sixty-four experts with research and/or clinical experience in medical care for people with IDD from a wide variety of countries were invited through email and asked to forward the invitation to potentially relevant participants (snowballing). Potential participants were identified from: (1) members of the International Association for the Scientific Study of Intellectual and Developmental Disabilities (IASSIDD) Health SIRG (special interest research group), (2) members of the IASSIDD Comparative policy and practice SIRG, (3) members of the GATE (Global Cooperation on Assistive Technology) community, (4) the network of the research team, and (5) international authors of research articles about the medical care for people with IDD. Eligible participants received study instructions and personal credentials to enter the Concept Systems Groupwisdom™ project website.

Thirty-five eligible experts responded positively and enrolled. Of these, 25 completed the brainstorming phase (71.4%), 21 completed the sorting phase (60%), and 18 completed the rating phase (51.4%). These numbers are in line with the recommendations for CM studies [20–22]. Table 2 describes the participant characteristics. The brainstorming phase yielded statements from 25 participants from 17 countries across six continents. On average, participants had 12 years of research experience and/or 17 years of clinical experience. Fifteen participants (43%) had experience in both research and clinical practice.

**Table 2:** Characteristics of participants

	**Brainstorming (*n* = 25)**	**Sorting (*n* = 21)**	**Rating (*n* = 18)**
**Continent of origin**			
Europe	7	8	8
Asia	6	4	3
North America	5	3	2
South America	2	2	2
Oceania	3	2	2
Africa	2	2	1
**Profession**			
Medical doctor	9	7	7
Allied health professional	7	6	3
Registered nurse	3	3	3
Other	6	5	5
**Years of clinical experience**			
0–5	4	4	2
6–10	4	1	2
11–15	5	2	1
16–20	4	2	3
> 20	8	12	10
**Years of research experience**			
0–5	6	4	3
6–10	6	2	2
11–15	8	9	9
16–20	2	2	1
> 20	3	4	3
**Sampling plan**			
Network of research team	6	4	4
IASSIDD Special Interest Research Groups (SIRGs)	5	4	4
GATE community	2	3	2
Snowballing	11	10	8
Abstract books of previous congresses	1	0	0

### Data collection and analysis

Preparation, data collection, and data analysis repeatedly alternated in nine consecutive steps (Table 1). Online data collection (step 2 and 5) took place from July 2020 through November 2020.

In step 1, the brainstorming phase was prepared by development of a recruitment plan and the focus prompt, which is a statement that participants respond to. During the (online) brainstorming phase (step 2), participants individually completed the following focus prompt in as many ways as possible: ‘If you asked me to describe medical care for people with IDD in my country, I would say…’. Participants were encouraged to think about medical care for people with IDD in its broadest sense and to consider different experiences or information relating to the focus prompt. They could enter an unlimited number of responses and could view all responses previously generated by different participants to stimulate their thinking process [19]. Demographics on country of residence, profession, years of research and/or clinical experience, and permission to be mentioned in the acknowledgements were obtained.

In step 3 (qualitative analysis of raw statements), three members of the research team individually read and coded each raw statement looking at different types of statements formulated by participants (rather than content related to medical care). Next, the researchers compared their analyses and collaboratively decided on three main types of statements (descriptive, normative, and prescriptive). Examples of these types of statements are provided in part 3.1 of the Results section.

In step 4 (preparation organization phase), four research team members prepared the final statement list for sorting and rating in step 5 by deleting duplicates, ensuring that each statement represented a unique idea, and light editing for clarity.

In step 5, the organization phase, statements were sorted by participants individually into groups of similarly themed statements and participants created descriptive labels for each group based on what their unifying content. Next, participants individually rated each statement on a 7-point Likert scale based on its importance for the concept of medical care for people with IDD (1 = relatively unimportant; 7 = extremely important). To include as many different views as possible, participants did not have to finish the brainstorming phase to participate in the organization phase. The raw data from each step are available upon request.

In step 6 (preparation analysis phase), three participants’ sorting data were excluded because of incompleteness (75% or fewer statements were sorted) or inaccurate sorting (misinterpretation of sorting assignment; e.g., application to one’s own country).

In step 7 (concept mapping analysis), the sorting data of the remaining participants were used to create a similarity matrix showing how frequently participants sorted the same statements together. Multidimensional scaling, a technique that plots each statement as a point on a map, was used to create a 2D point map. Statements participants more frequently sorted in the same pile were plotted closer together on the point map, with spatial distance between each point representing how often statements were sorted together. To determine how well the 2D point map fitted the original sorting data, a stress value was calculated: a lower stress value suggests a better overall fit [23].

Hierarchical cluster analysis was used to combine spatially close statements into clusters. Bridging values (range 0–1), defined below, were calculated for all statements in the possible cluster solutions to help interpret the clusters and select the final cluster solution. A lower bridging value indicates that a statement was frequently sorted with statements adjacent to it. Cluster sizes and names were determined by the procedure recommended by Kane and Trochim (Table 1; step 7) [23]. The final cluster names were checked on language and connotation by one native English-speaking participant. Finally, participants’ importance ratings were averaged for each statement and per cluster.

Sensitivity to sampling or robustness of the concept map (step 8) was investigated with a Jackknife procedure [24]. The Jackknife procedure entailed performing the CM analysis (step 7) 18 times on a sample of 17 participants, systematically omitting one participant (n-1). The 18 resulting concept maps were compared with the concept map resulting from the full data (including all 18 participants). The number of statements placed in a different cluster and the number of statements leaving and entering the cluster were calculated and it was assessed whether the statements within the cluster covered the same theme as in the original allocation using full data.

In step 9 (qualitative analysis of final cluster map), the final cluster map was scrutinized for overarching conceptual themes that could point towards an underlying dimension within the data beyond the scope of the used software.

## Results

### Qualitative analysis of raw statements (step 3)

Brainstorming (step 2) resulted in 92 raw statements (see Additional file 1). The qualitative analysis of these raw statements indicated that there was a large diversity in how respondents interpreted the focus prompt; most raw statements were formulated negatively, and since statements were formulated from a specific country context, they could contradict each other. For example: *“Quality of services for people with IDD in hospitals or at GP’s is usually low”* and *“Global standards for medical care are high. Therefore, most of the people with IDD get adequate treatment”*. Although the focus prompt of this study guided towards descriptions of medical care, the collected statements show that participants did not always make a clear distinction between medical care and health care.

The qualitative analysis categorized raw statements into three main response types: 1) descriptive responses describing current medical care for people with IDD, for example: “*There are guidelines and tools to support health care providers for this group”*, 2) normative opinions about what good medical care for people with IDD should entail, for example: “*Do any of us have a nurse following us around to pass medications when we are out of our home? Why is this normal for IDD population?*”, and 3) prescriptive personal opinions and aspirations on what medical care for people with IDD should look like, for example: “*Improving communication between medical practitioners, allied health, and support organizations would improve medical care for people with IDD in my country”*. Often, a statement combined two or more of these types. An example of a raw statement which contains descriptive as well as normative and prescriptive elements is: *“During Covid we find that we are essential workers. We are not paid that way (descriptive), how can we provide high quality care when we cannot attract high quality workers with the salary the state funds (prescriptive)? Our population suffers the most (normative)”*.

### Concept mapping analysis (step 7)

#### Point map

To prepare for the organization phase (step 5), the 92 raw statements were synthesized to 79 final statements (after removal of 13 duplicates) (see Additional file 2). Participants sorted the statements in a minimum of four and a maximum of 18 clusters. Based on the sorting data (acquired in step 5), a point map was created, in which the proximity of the 79 statements to one another is projected on a 2D map (see Additional file 3).

#### Final concept map

**Fig. 1:**
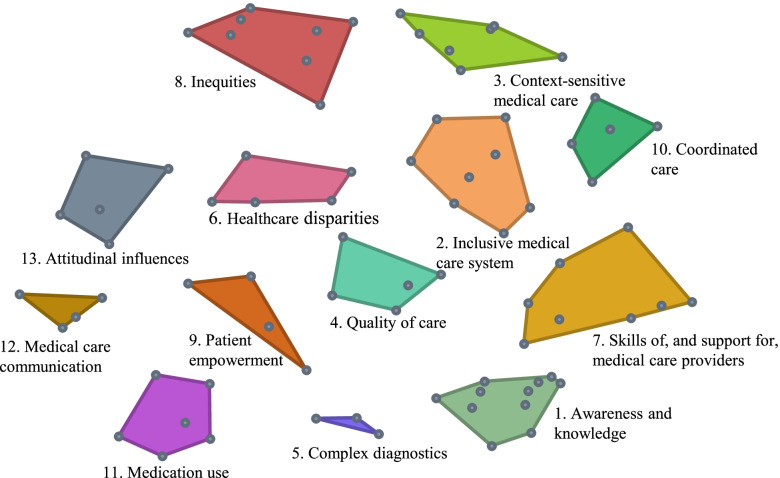
Final concept map: a spatial representation of how the statements (dots) relate to the clusters

The final concept map consists of 13 clusters. Figure 1 illustrates how the 79 statements (dots) are spatially located within the clusters. The closer the statements and clusters are together, the more they are related to one another. The stress value of the final concept map is 0.28, which matches other CM projects in which stress values range between 0.21 and 0.37 [19]. A lower stress value suggests a better overall fit of the concept map to the sorted statements.

The 13 clusters (Table 3) represent different aspects used to describe medical care for people with IDD. For each cluster, a description was formulated by the research team based on the statements within the clusters and informed by literature. Cluster bridging values range between 0.12 and 0.69, representing an overall moderate level of cluster anchoring. The four clusters, *Awareness and knowledge* (0.12), *Inclusive medical care system* (0.16), *Context-sensitive medical care* (0.21), and *Quality of care* (0.21) have statements with relatively low mean bridging values, indicating that these statements were frequently sorted with nearby statements. The *Attitudinal influences* cluster (0.69) has a relatively high mean bridging value.

**Table 3:** The 13 clusters and their descriptions, mean bridging values (B), and importance ratings (I)

**Cluster (number of statements)**	**Description**	**B**^a^	**I**^b^
1. Awareness and knowledge [11]	Specific awareness about, and knowledge of, the health needs and problems of people with intellectual and developmental disabilities (IDD) is essential in their medical care	0.12	5.4
2. Inclusive medical care system [8]	The medical care system has to make reasonable adjustments to accommodate persons with IDD and their specific health needs [25]	0.16	5.4
3. Context-sensitive medical care [7]	The organization and funding of medical care for people with IDD differs between and sometimes within countries, for different age groups (children and adults), and compared with the general population	0.21	5.3
4. Quality of care [5]	The quality of medical care for people with IDD differs between countries and preventive care is often lacking	0.21	5.1
5. Complex diagnostics [3]	People with IDD often cannot verbalize their complaints/symptoms and/or have unidentified conditions, making diagnosis complex	0.26	5.5
6. Healthcare disparities [5]	The access, use, and quality of medical care differs between people with IDD and the general population [26]. For example, their needs are not adequately recognized and addressed, they experience communication difficulties, and they are subject to restrictive rules	0.28	4.9
7. Skills of, and support for, medical care providers [8]	Medical care providers need specific skills and preconditions (e.g., time, guidelines, tools, resources to facilitate collaboration between sectors) to support the unique health and social considerations of people with IDD	0.29	5.4
8. Inequities [8]	People with IDD do not have the same opportunities concerning medical care access, use, and quality compared with the general population. These differences, on for example the socioeconomic, geographic, and racial level, are avoidable [26]	0.30	5.5
9. Patient empowerment [4]	People with IDD are fully empowered when they have sufficient knowledge to make rational decisions, sufficient control and resources to implement their decisions, and sufficient experience to evaluate the effectiveness of their decisions concerning their medical care [27]	0.31	5.5
10. Coordinated care [5]	Medical care for people with IDD needs to be well-coordinated because many levels of care (providers) are included. This especially concerns horizontal collaboration, such as partnerships and networks within and between sectors and collaboration between health professionals [28]	0.35	5.5
11. Medication use [6]	Overmedication is common in the medical care for people with IDD, and people with IDD need to be properly informed about, and consent to, their medication	0.40	5.1
12. Medical care communication [4]	People with IDD are often supported by others in their health communication and in making medical care decisions [8]	0.54	5.7
13. Attitudinal influences [5]	The medical care use of people with IDD is affected by attitudinal influences: assumptions that discriminate against people with IDD [29]. Because of these attitudinal influences, people with IDD are labelled, stigmatized, and not always adequately protected	0.69	5.0

^a^B = bridging value between 0 and 1 (a lower bridging value indicates that the statements within this cluster were frequently sorted with statements immediately adjacent to it)

^b^I = importance rated on a 7-point Likert scale

#### Importance ratings

The importance ratings of the statements (acquired in step 5) ranged from 3.3 to 6.2 (see Additional file 2 for the importance ratings of all statements). Table 3 shows that there was limited variability in ratings of importance across clusters. On average, the statements within the *Healthcare disparities* cluster (4.9) were rated least important and the statements within the *Medical care communication* cluster (5.7) were rated most important.

### Sensitivity of the concept map (step 8).

Comparison of the Jackknife trials (n-1) with the original concept map shows that, on average, 16 of the 79 statements (range: 4–27) were placed in another cluster, most often a cluster nearby. The clusters representing *Inequities, Context-sensitive medical care, Medication use*, and *Awareness and knowledge* were present in all Jackknife trials. In contrast, the clusters *Attitudinal influences* and *Patient empowerment* did not appear in at least one-third of the Jackknife trials, meaning that their presence is sensitive to sampling variation. Merging these clusters with the clusters *Quality of care* and *Medical care communication* in an 11-cluster solution reduced the cluster map’s sensitivity to sampling variation. However, this would have excluded the clusters *Patient empowerment* and *Medical care communication*, which provide relevant information, especially for the patient perspective. Therefore, we retained the original 13-cluster concept map.

### Qualitative interpretation of the concept map (step 9).

**Fig. 2:**
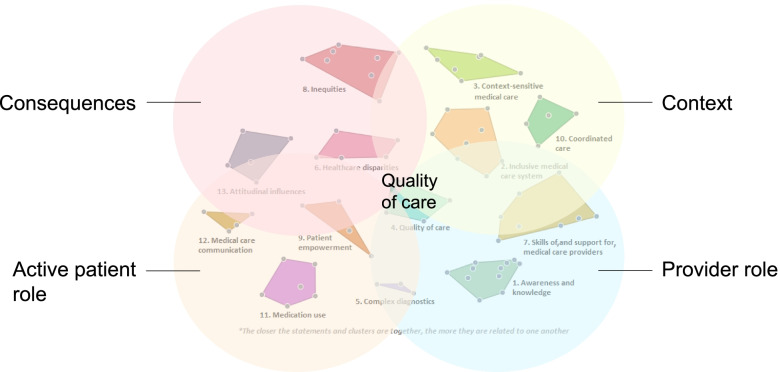
Five themes characterizing medical care for people with intellectual and developmental disabilities

The 13 identified clusters can be grouped in five overarching themes: (i) the active patient role, (ii) the provider role, (iii) the context of care, (iv) the consequences of care for people with IDD, and (v) quality of care (Fig. 2).

First, the active patient role appears in cluster 9, 11, and 12. Cluster 9: *Patient empowerment* reflects a lack of preconditions to empower people with IDD to participate in medical care decisions, such as health literacy and prolonged consultations. Cluster 11: *Medication use* addresses practical problems in medical care for people with IDD, such as overmedication and lack of consent for medication. Cluster 12: *Medical care communication* indicates that there are communication challenges for people with IDD and that communication support is important.

Second, the role of medical care providers is represented in clusters 1, 5, and 7. Cluster 1: *Awareness and knowledge* is the largest cluster. The statements within this cluster indicate that medical care professionals need adequate knowledge, experience, and understanding of the medical care needs of people with IDD. Cluster 5: *Complex diagnostics* addresses communicational and behavioral challenges that complicate diagnosing medical conditions in people with IDD. Cluster 7: *Skills of, and support for, medical care providers* points to the specific skills and preconditions needed by medical care providers to provide medical care for people with IDD.

Third, the influence of the context on medical care for people with IDD appears in cluster 2, 3, and 10. Cluster 2: *Inclusive medical care system* addresses lack of accessible medical care because of fragmentation and unpreparedness of care systems regarding special needs of people with IDD. Cluster 3: *Context-sensitive medical care* indicates that context influences medical care for people with IDD; the organization of this care differs for instance between countries, but also between age groups (children/adults). Cluster 10: *Coordinated care* reflects the multidisciplinary character of medical care for people with IDD and the need for coordination between medical care providers.

Fourth, the consequences of current medical care for people with IDD are presented in cluster 8, 13, and 6. Cluster 8: *Inequities* addresses the perceived unfair inequality in opportunities of people with IDD in medical care. Cluster 13: *Attitudinal influences* indicates that the medical care use of people with IDD is influenced by discriminatory assumptions, such as stigmatization, labelling, and inadequate protection. Cluster 6: *Healthcare disparities* shows that there are differences in access, quality, and use of medical care for people with IDD compared with the general population.

Fifth, the quality of medical care for people with IDD is represented in cluster 4: *Quality of care*. This cluster shows that the quality of medical care for people with IDD differs between countries and preventive care is often lacking. The Quality of care cluster is situated in the middle of the concept map, indicating that it relates to all four themes around it.

## Discussion

The additional analysis of the raw statements (step 3) showed that participants described medical care for people with IDD using statements that reflect current medical practice, their ideas on good practice, and aspirations for future medical care for people with IDD. Furthermore, statements could contradict each other because multiple international perspectives were included. This underlines the need for a framework to improve international understanding of medical care for people with IDD that specifically addresses IDD related themes, as this study identifies in its concept map. The developed concept map includes 13 aspects that characterize medical care for people with IDD and that can be grouped in five overarching conceptual themes: *1) active patient role, 2) role of medical care providers, 3) influence of context, 4) consequences of current arrangements, and 5) quality of care.* These themes provide an explorative overview of medical care for people with IDD, and indicate that this care goes beyond the practical medical care provision between providers and patients.

Active patient involvement is an important aspect of medical care for people with IDD. Although this aspect is highlighted in existing models of medical care, communication difficulties of people with IDD and negative attitudes towards people with IDD may hinder active involvement in their medical care (decisions) [30]. People with IDD need support in communicating health problems and adequate information when making medical care decisions, and the involved medical care professionals need to adequately exchange health information and coordinate actions [31, 32]. The patient should be given the space and modes of communication to express their needs. In line with this, Mastebroek et al. [33] identified perceived barriers and facilitators for the health information exchange between medical care providers and people with IDD. Medical care providers can be well placed to facilitate the shared decision making with people with IDD [34].

Because of the complexity of diagnosing the medical care problems of people with IDD, many different professionals are involved in the medical care of people with IDD. Lack of coordination between these professionals can for example lead to overmedication or unnecessary or duplicate testing [35]. Integrated care initiatives can potentially lower fragmentation in the medical care for people with IDD, but this has not been demonstrated empirically due to lack of clear definitions and the difficulty of applying disease-specific initiatives to IDD [35]. Many people with IDD have underlying conditions that are not identified due to behavioral or communication challenges and challenges in presentation [30]. Despite the increasing knowledge on the needs for specific skills and knowledge in medical care providers [11, 36], authors of recent review articles agree that medical care for people with IDD still lacks adequate support [9–11, 37, 38]. This lack can possibly be addressed by systematically increasing the attention/priority for the special medical care needs of people with IDD in medical curricula and education [39, 40].

While similar preconditions are recognized internationally, the context and organization of medical care differs between and sometimes within countries and for different life stages [9, 14, 15]. The international differences are also underlined in this study by the contradicting statements. The different international responses to the still ongoing COVID-19 pandemic have shown that understanding the context in which medical care for people with IDD is provided is important to learn from other countries and improve the medical care for people with IDD [41]. There is a need for a better understanding of the different international models of medical care for people with IDD [10]. This study’s concept map provides an exploration of themes and aspects that are relevant in medical care for people with IDD complementary to the already existing models of medical care, which can be used as a basis for future studies that can further elaborate on these themes and aspects.

### Limitations

This study’s findings should be interpreted in light of the following limitations. This study included 25 participants. This number is in line with the recommendations for CM studies [20–22]. Given the exploratory and international nature of this study, sampling focused on heterogeneity of participants and snowballing allowed us to recruit a wide diversity of participants, originating from 17 countries and six continents. The participants provided us with the opportunity to set up a first exploration of which aspects constitute the concept of medical care for people with IDD. Nevertheless, future studies with larger respondent groups will be needed to further elaborate on these aspects.

This study reflects only expert researchers’ and practitioners’ perspectives on medical care for people with IDD and lacks perspectives of people with IDD themselves and their caregivers. Explicating concepts is helpful for people with IDD to share their perspectives on complex topics [42], and the themes and clusters identified in this study can provide a basis for people with IDD to be actively involved in future studies.

Although the CM approach has been widely applied in health care research and is suitable to disentagle complex phenomena into more simple individual components [43], it requires further critical assessment. For example, the quality of results is often assessed based on the stress values of previous empirical studies [44]. However, no clear standards for acceptable stress values have been established analytically. Péladeau and Dagenais [44] contend that reversing the order of multidimensional scaling followed by hierarchical cluster analysis has major advantages over the original order. Because the software used was limited to the original order of analysis, this study assessed additional sensitivity to sampling variation through a Jackknife procedure, which has been applied in a similar study before [45]. Moreover, we opted to add additional qualitative analyses of the raw statements and of the final cluster map to better understand and represent the collected data and prevent loss of information. The additional dimension in answer types (current medical practice, ideas on good practice, and aspirations for future medical care for people with IDD) is information that would have been lost in the traditional concept mapping procedure.

The CM software allowed for international participants to contribute to this study online. However, language might have been a barrier for non-native English speakers. Also, nuances in meaning may have been lost in translation between formulating statements and interpretation by other participants and the research team. However, the CM method was suitable to provide a first exploration of the concept of medical care for people with IDD.

This study aimed to explore themes and aspects of medical care for people with IDD. The collected statements show that participants did not always make a clear distinction between medical care and health care. This highlights the interrelatedness of both concepts, also within IDD specific care.

During the CM procedure, the research team made decisions on how to proceed. These decisions included evaluating the sorting and rating data and choosing the number of clusters in the final cluster map. For transparency, these processes were described in the methods section; detailed information is available upon request. We recommend future studies to also include this transparency and an additional qualitative analysis of the collected responses to better understand and represent the collected data and prevent loss of information.

## Conclusions

By providing an explorative overview, this study can serve as a first step towards an improved understanding of the concept ‘medical care for people with IDD’. This study shows that the medical care for people with IDD is described as encompassing more than only the practical medical care provision between providers and patients. Themes such as active patient involvement, coordination of care, contextual influences on the medical care, and consequences of the medical care are also important themes to consider. The themes, clusters and statements identified through this study move beyond objective and countable data and help to provide additional content and context for the specific patient group of people with IDD to the dimensions of previous models of medical care. Future studies can further elaborate on these themes.

## Supplementary Information

**Additional file 1: Supplemental Digital Content 1.** The 92 unedited responses to the focus prompt.

**Additional file 2: Supplemental Digital Content 2.** Overview of statements per cluster with associated bridging values (B) and mean importance (I).

**Additional file 3: Supplemental Digital Content 3.** Point map visualizing the relationship and proximity of statements to one another.

## Data Availability

This study generated three datasets: the statements generated from brainstorming, the sorting data, and the participant rating data. Furthermore, the authors recorded a detailed description of the process undertaken during analysis. All datasets used during the current study are available from the corresponding author on reasonable request.

## Abbreviations

IDD: Intellectual and developmental disabilities
CM: Concept mapping
