# Supplementary material for: Exploring views on medical care for people with intellectual disabilities: an international concept mapping study
Source: Int J Equity Health. 2022 Jul 19;21:99. doi: 10.1186/s12939-022-01700-w (PMC9295354; doi:10.1186/s12939-022-01700-w)
Supplement: Supplementary file 1 — Additional file 1: Supplemental Digital Content 1. The 92 unedited responses to the focus prompt. [file 12939_2022_1700_MOESM1_ESM.pdf]

Supplemental Digital Content 1: The 92 unedited responses to the focus prompt.

Raw statements	Focus prompt: If you ask me to describe medical care for people with intellectual and developmental disabilities (IDD) in my country, I would say...
1	Private public participation
2	Overmedication is a problem for many with an IDD. This may be a problem in families too, but especially present in long term care facilities (most residents are on medication prescribed by psychiatrists). Lack of consent is a problem + side effects!
3	Poverty is a huge problem and medical care and health is closely related to poverty. Most people with IDD are likely to live in households with low income.
4	Prevention is lacking for people with IDD. Medical staff &parents report that many people with an IDD go to their GP/hospitals too late. This is probably due to stigma & previous bad experience + lack of services (e.g. too far from where they live).
5	There is a scarcity of practices that accommodate the special communication needs of people with IDD. Augmentative or alternative communication (AAC) options are not available at health services. Medical staff wouldn't even know how to use AAC.
6	Medical care for pple with an IDD in Hungary is largely underfunded. There is no training for medical professionals about the special needs of people with IDD. Thus, quality of service at hospitals or GPs is usually low for people with IDD.
7	In JAPAN, Decision Support for the people who have IDD, especially in medical field, is a problem nowadays. Who has a right to decide to take medicine, to accept surgery, on behalf of them? Family members, caretakers bear the responsibilities.
8	In Japan I can find more specialists has come to understand people with IDD nowadays. Every doctor has different values and I think it is important to communicate thoroughly with specialists by the primary care doctors for people with IDD.
9	It is completely under funded
10	In Canada, adults with IDD access general, publicly funded health care. There remains many people in the healthcare workforce that do not feel that they have the competencies to support the unique health and social considerations of this group.
11	There are guidelines and tools to support health care providers for this group

12	In Canada, medical care for adults with IDD is not well coordinated between primary and specialist care and many individuals do not have access to comprehensive team-based primary care.
13	In Canada, there remain many adults with IDD that do not receive recommended preventative care and have underlying conditions that are not identified secondary to behavioural/communication challenges and challenges in presentation.
14	Do any of us have a nurse following us around to pass medications when we are out of our home? Why is this normal for DD population?
15	Med Rule that allows DSPs pass medications is too restrictive and doesn't allow for residents to travel much less attend special olympics without a nurse to travel with them to pass meds to them. What facility has this kind of money?
16	our folks are being pushed into MMAI programs which require referrals for specialists, many not in the immediate area. Strains resources to get them to appointments and delays care. They lose their current PCP and forced to see another.
17	medical care is strained by providers who do not understand that our folks don't understand their questions and./or are poor reporters...they must use staff to fill in the gaps of information but often ignore staff, prompting unnecessary testing.
18	During Covid we find that we are essential workers. We are not paid that way, how can we provide high quality care when we can not attract high quality workers with the salary the state funds? Our population suffers the most.
19	DD diagnosis does not allow for them to enter nursing home to live out their lives...they can NEVER retire also must do programming no matter their age or progressing medical conditions. This is NOT normalization. Once 65+ why choices gone?
20	Approaches that aim to increase self determination of the person with ID, such as shared decision making and supported decision making are promising practices, but they are only being used by few people in small pilot areas.
21	Families and people with ID have significant challenges with transition from pediatric medical care providers. There are few capable medical care providers for adults in many areas.
22	In the US, there is not agreement about the best approach - whether it is to established specialized care centers or whether it is better to support all clinicians to provide good integrated care.
23	People with with ID in the US generally do not have access to specialized clinicians who have particular knowledge and training.
24	There are geographic, socioeconomic and racial inequities in access to medical care for the population as a whole.

25	In Japan, medical care is defined as a part of medical treatment which is provided by non-medical staff or family members of people with IDD at their homes or schools, other than in medical hospitals.
26	There is specialized education for people with IDD, but not medical specialty in IDD in Japan. However, we do have a category called Ryoiku in which people with PIMD are cared for their medical, healthcare and welfare issues.
27	The specialist skills to assess and provide treatment for people with IDD is not acknowledged as a specialty, like cardiology or neurology. It is a specialist skill to understand the comprehensive health needs of people with IDD.
28	There is no financial incentive for adequate healthcare to be provided by medical staff, it is usually only those with an interest in the area work in this space.
29	People with IDD have multiple barriers to accessing the healthcare they need. Medical staff often lack the expert knowledge to assess and manage health needs, communication is insufficient, not enough education available, rights of IDD not acknowled
30	Several reports in Norway on Health status and health monitoring of adult people with ID, have for many years told us that it is not sufficient. Care workers do not capture signals of diseases soon enough to be able to treat.
31	In Norway, we lack both general- and acute health knowledge among care workers who work with people with ID.
32	Ageing and Health has developed a Hospital passport for people with ID to bring when treated in hospitals or other units.
33	All habitants in Norway have a general practitioner ,and people with ID are entitled a yearly health check by their GP. But not all of them receive a Health check due to lack of resources.
34	In Norway we have habilitation services that offermultidisciplinary inpatient and outpatient services for people with ID. Children and adult care are separated.
35	In Switzerland, special needs of PWID, especially adults, and their care have to be recognized and adequately been reimbursed.
36	There are few specialized units in Switzerland available for people with ID.Global standards of medical care is high, therefore most of PWID get adequate treatment. However: care in the community and for challenging behaviour must be improved.
37	A particular issue arises at transition from paediatric to adult care- as children with IDD are surviving longer there is an issue with who provides ongoing care for them - adult practitioners are not used to looking after them in the same way

38	There is an awareness of the increasing need to support people with IDD in Australia. However there are still many misconceptions about their needs and the complexity of their conditions.
39	In Israel I believe that the medical and developmental services are all in all very good for kids. However, I think that there is a gap between their services and the ones that are offered to adults.
40	Average life span in most west African countries rangea from 35-50 so when you have an IDD life span is even shorter due to a lot of factor becauae the quality of life and healthcare is completely beliw standard for people with IDD.
41	The most vulnerable are not always the most protected so it makes a really difficult situation even worse when you are located in the rural setting because there is inadequate healthcare and inequalities of care
42	Chikdren suffer a lit especially once with IDD, YHEY do not fit the box and are labelled in the society
43	The rural are is the most deprived due to lack of accessibility to healthcare and poor road networks
44	Iits needs structure and dedication
45	The dissability bill has just been signed into law and this took so long but up to now it is not being enforeced
46	People with IDD their needs are never met and no formal provision to meet their needs i.e some hospitals dont even have ramps etc, people are not being treated with dignoty or informed properly regarding their diagnosis
47	Medical care for people with IDD is very appalling in my country and there are 2 types of medical care:the Public owned by the government and the private owned by individuals or group of individuals. The governement healthcare is divided into 3 tiers
48	Medical care for people with IDD is very appalling in my country and there are 2 types of medical care:the Public owned by the government and the private owned by individuals or group of individuals. The governement healthcare is divided into 3 tiers
49	Most ID persons can only get medical care if their support workers or carers decide they need it and seek it out for them. There are no clear / simple ways for person with ID to reach out to GP if they feel they need it.
50	If the ID person cannot verbally communicate and articulate what is wrong, then treatment is delivered 'around' them - directed at whoever is supporting them rather than the person themselves

51	Most medical providers don't seem to understand the impacts of complex disabilities or how to support the conversations which need to take place for their appropriate care
52	It is not readily accessible and where it is accessible, there is stigma attached to it so few people will willingly seek treatment from the facilities providing medical care for people with IDD
53	Psychotropic drugs are used too often in the medical care of people with IDD in my country.
54	It would be good to reduce the medicalisation of medical care for people with IDD in my country
55	Medical care for people with IDD is not given enough prominence in my country.
56	Improving communication between medical practitioners, allied health, and support organisations would improve medical care for people with IDD in my country.
57	Medical care for people with IDD in my country is fragmented.
58	Some children with IDD do not have access to medical care as it is not considered as priority since it is not life threatening.
59	Most children with ID are first suspected by their parents and neighbors/ community members especially in rural area. In urban, they are first suspected by class teacher in nursery or school especially for those children with ID with working parents.
60	There is a use of both indigenous and western knowledge in the diagnosis of ID. Conditions of children are often suspected by parents especially those in rural areas using indigenous knowledge
61	Most children with ID who don't present any other clinical condition, are first suspected by their parents, mainly when they have older siblings, or by teachers on daycare and school. Physicians seem more alert to severe than mild conditions.
62	Children with genetic conditions coursing with ID tend to get earlier diagnose and treatment. Those who fall out of this, eg ASD, mild CP and others, are less likely to get invloved in early intervention programs timely.
63	Overall it varies greatly from one region to another, but most states and cities rely on a unprepared helth care system when it comes to diagnose and the education system mix inclusive and segregate options.
64	regarding ID helath care there is need for a huge effort of all stakeholders to come to knowledge transfer, collaboration and integrated care between the different health care sections

65	we are lucky to have a specialized training on ID medicine. We are not lucky that there are barriers between the ID specialized care and primary care and between ID specialized care and mental health care.
66	4) Establish in which countries there are specifically courses dedicated to IDD in the health careers at university. If there are plans in this knowledge for pregrade students, and the number hours wich is dedicated to IDD by students. Much of this
67	3) Establish the percentage of schools dedicated to IDD, in relation to normal schools, in the different countries.
68	of their funds and how they are used in general.This would allow establishing regional comparative indices of concern for IDD over time.
69	the responsible administrator for these programs, if they exist. 2) Establish an International voluntary list of Governmental and Non-Governmental Organizations that are dedicated to supporting IDD in the different countries, indicating the source
70	1) Establish a comparative document that lists the laws with which the different States have assumed the reality of people with IDDWith a table that includes the percentage of GDP dedicated to support disability, its national geographic distribution
71	Most providers do not understand the complexity of their care. They are used to speaking directly with the patient and many of our residents have IQ of small children or cannot verbalize or answer questions accurately.
72	People with IDD within disability support in the municipality do have some medical support specialized in IDD (nurses, occupational and physio -therapists), however without primary health care competence - possible leading to unequal care in a way
73	Primary health care available for all citizens, exist on 300 places in a 2 million city such as Stockholm, with a lot of doctors on each place in turn meeting to few individuals with IDD to get experience enough to see pattern connected to the IDD
74	Although information about all different familiar syndromes is available on governmental webpages health care professionals seldom have the time (or knowledge about the need) to look into and learn about different syndromes comorbidity
75	The education system for doctors as well as nurses have very little education in this populations needs
76	In Sweden similar to Canada children and adolescents with IDD are mostly well served from the health care organizations. It is after that, when they have to find help in the general health care system that it gets problematic.
77	Knowledge is very scarce within the health care system among what might follow an IDD concerning difficulties such as understanding verbal information or the need for extra time to be able to communicate needs and questions.

78	just as unsure with regards to health insurance as it is for the general population, but with higher health care use/needs
79	not enough time (more time needed than usual) to involve the person with ID in decision-making
80	lack of specific knowledge on ID in health care professionals
81	depending on available expertise in geographical area
82	not accessible enough
83	in silos
84	it is very much multidisciplinary
85	it is still influenced by stigma
86	it does not matter if you live in a high income country or a lower income country, people with ID always depend in some way on the people around them, their care system, to signal needs and to have access to healthcare.
87	lack of awareness and knowledge on the health needs of people with ID and how you can signal and assess those needs.
88	it needs to be more proactive in order to reduce health inequity and underdiagnoses and with that improve quality of life. The ID physician is trained to do so.
89	very heterogenous and different per country and even within one country you find different standards, approaches etc
90	When someone with ID is in hospital, if they have family caregivers they can have someone to advocate for them, but if their family has passed or is not involved, they may struggle having advocates in hospitla.
91	In Canada, children and teenagers have good supports as long as they still go to school, but once they finish secondary school it can be hard to access care.
92	In Canada, it varies a lot by different provinces. Some provinces have very good medical care for people with ID, others not so much.
