# Supplementary material for: Exploring views on medical care for people with intellectual disabilities: an international concept mapping study
Source: Int J Equity Health. 2022 Jul 19;21:99. doi: 10.1186/s12939-022-01700-w (PMC9295354; doi:10.1186/s12939-022-01700-w)
Supplement: Supplementary file 2 — Additional file 2: Supplemental Digital Content 2. Overview of statements per cluster with associated bridging values (B) and mean importance (I). [file 12939_2022_1700_MOESM2_ESM.pdf]

**Supplemental Digital Content 2: Overview of statements per cluster with associated bridging values (B) and mean importance (I)**

| ID | Statement | B* | I† |
|---|---|---|---|
| | **Cluster 1: Awareness and knowledge** | 0.12 | 5.4 |
| 5 | There is an awareness of the increasing need to support people with IDD | 0.324 | 5.4 |
| 7 | Most medical care providers do not understand the impacts of complex disabilities | 0.034 | 5.7 |
| 8 | Medical care providers are not aware of the need to learn about different syndromes | 0.005 | 5.5 |
| 11 | There is a lack of both general and acute health knowledge and skills among care workers who work with people with IDD | 0.082 | 6.0 |
| 13 | The education system for health care professionals and medical professionals has very little education in the needs of people with IDD | 0.071 | 6.1 |
| 14 | There is a lack of knowledge on the health needs of people with IDD and how to signal, assess, and manage those needs | 0.055 | 5.9 |
| 16 | Medical care providers meet too few individuals with IDD to get enough experience to see patterns connected with IDD | 0.000 | 5.4 |
| 19 | Most medical care providers do not seem to understand how to support the conversations which need to take place for appropriate care | 0.106 | 5.6 |
| 36 | Medical care providers must use staff to fill in the gaps of information but often ignore staff | 0.134 | 4.9 |
| 62 | More specialists have come to understand people with IDD nowadays | 0.113 | 5.4 |
| 75 | There is use of both indigenous and western knowledge in the diagnosis of IDD | 0.431 | 3.3 |
| | **Cluster 2: Inclusive medical care system** | 0.16 | 5.4 |
| 10 | People with IDD generally do not have access to specialized clinicians who have particular knowledge and training | 0.199 | 5.9 |
| 35 | Many people with IDD do not have access to comprehensive team-based primary care | 0.108 | 5.5 |
| 37 | Medical care for people with IDD is fragmented | 0.162 | 5.6 |
| 38 | Most states and cities rely on an unprepared health care system when it comes to diagnose | 0.203 | 5.3 |
| 43 | Medical care for people with IDD is not given enough prominence | 0.106 | 5.8 |
| 53 | There are few specialized units available for people with IDD | 0.140 | 5.4 |
| 64 | The organization of the medical care system delays care | 0.156 | 5.6 |
| 74 | Medical treatment is only provided by non-medical staff or family members of people with IDD | 0.190 | 4.4 |
| | **Cluster 3: Context-sensitive medical care** | 0.21 | 5.3 |
| 2 | Medical care for people with IDD varies greatly between countries and from one region to another | 0.253 | 5.8 |
| 32 | There is a gap between the services offered to kids and adults: they are very good for kids | 0.260 | 5.8 |
| 40 | Medical care for people with IDD is underfunded | 0.158 | 5.6 |
| 41 | There is no financial incentive for adequate healthcare provision for people with IDD by medical staff | 0.192 | 5.4 |

| | | | |
|---|---|---|---|
| 48 | Medical care for people with IDD is just as unsure with regards to health insurance as it is for the general population, but with higher health care use/needs | 0.229 | 4.7 |
| 55 | Adults with IDD access general, publicly funded medical care | 0.199 | 5.5 |
| 56 | There are two types of medical care: the public, owned by the government, and the private, owned by individuals or groups of individuals | 0.155 | 4.1 |
| | **Cluster 4: Quality of care** | 0.12 | 5.1 |
| 44 | Prevention and proactive care is lacking in medical care for people with IDD | 0.100 | 5.9 |
| 49 | Quality of services for people with IDD in hospitals or at GP's is usually low | 0.096 | 5.2 |
| 50 | Global standards for medical care are high. Therefore most of the people with IDD get adequate treatment | 0.099 | 4.3 |
| 51 | Medical care for people with IDD is completely below standards | 0.180 | 4.7 |
| 65 | Medical staff and parents report that many people with IDD go to their GP/hospital too late | 0.148 | 5.2 |
| | **Cluster 5: Complex diagnostics** | 0.26 | 5.4 |
| 20 | Medical care providers are used to speaking directly with the patient, but many people with IDD have a low IQ and cannot verbalize or answer questions accurately, which is problematic | 0.301 | 5.7 |
| 58 | Many people with IDD have underlying conditions that are not identified secondary to behavioural/communication challenges and challenges in presentation (diagnostic overshadowing) | 0.215 | 5.6 |
| 59 | There is too much underdiagnosis | 0.260 | 5.0 |
| | **Cluster 6: Healthcare disparities** | 0.28 | 4.9 |
| 24 | There are no clear/simple ways for a person with IDD to reach out to a GP if they feel they need it | 0.195 | 5.4 |
| 42 | People with IDD, their needs, and their care need to be recognized and adequately be reimbursed | 0.344 | 5.8 |
| 66 | Children with genetic conditions coursing with IDD tend to get earlier diagnose and treatment. Those who fall out of this are less likely to get involved in early intervention programs timely | 0.191 | 5.4 |
| 76 | Rules that allow direct support persons to pass medications to people with IDD are too restrictive | 0.320 | 3.4 |
| 78 | Intellectual disability diagnose overrules retirement and nursing home care on old age | 0.374 | 4.5 |
| | **Cluster 7: Skills of, and support for, medical care providers** | 0.29 | 5.4 |
| 9 | There are few competent medical care providers to support the unique health and social considerations of people with IDD | 0.326 | 5.9 |
| 12 | Health care professionals seldom have the time to look into and learn about different syndromes | 0.040 | 5.6 |
| 33 | Medical care for people with IDD is very much multidisciplinary | 0.464 | 5.3 |
| 39 | There is a need for a huge effort of all stakeholders to come to knowledge transfer, collaboration and integrated care between the different healthcare sections | 0.611 | 5.8 |
| 52 | The specialist skill to assess and provide treatment for people with IDD is not acknowledged as a specialty, like cardiology or neurology | 0.147 | 5.2 |
| 54 | In some countries, there is a specialized training on ID medicine, however, these specialists do not have primary health care competencies | 0.211 | 4.6 |

| | | | |
|---|---|---|---|
| 63 | There are guidelines and tools to support health care providers for people with IDD | 0.312 | 5.5 |
| 73 | The values of the medical care provider influence the provided care | 0.239 | 5.5 |
| | **Cluster 8: Inequities** | 0.3 | 5.5 |
| 1 | There are geographical inequities in access to medical care for people with IDD | 0.134 | 6.0 |
| 45 | There are socioeconomic inequities in access to medical care for people with IDD | 0.219 | 5.9 |
| 46 | There are racial inequities in access to medical care for people with IDD | 0.294 | 5.5 |
| 47 | There is a lack of accessibility of medical care for people with IDD in rural areas due to, among other things, poor road networks | 0.221 | 5.3 |
| 60 | Medical care for people with IDD is closely related to poverty | 0.387 | 4.9 |
| 69 | There are inequalities of medical care for people with IDD | 0.208 | 6.0 |
| 70 | In rural areas, people with IDD are first suspected by their parents and neighbors/community members. In urban settings, they are first suspected by their parents or teachers on daycare and school | 0.691 | 4.7 |
| 79 | An overview of relevant laws, organizations, schools dedicated to IDD students, and funds is lacking | 0.243 | 5.3 |
| | **Cluster 9: Patient empowerment** | 0.31 | 5.5 |
| 15 | There is not enough education available for people with IDD | 0.314 | 5.6 |
| 23 | People with IDD are not treated with dignity in medical care provision | 0.267 | 5.2 |
| 61 | More time is needed to involve a person with IDD in medical decision making | 0.410 | 6.1 |
| 72 | Some people with IDD do not use medical services because of previous bad experiences | 0.243 | 5.1 |
| | **Cluster 10: Coordinated care** | 0.35 | 5.5 |
| 31 | Children and adult medical care are separated and transitions are problematic | 0.210 | 5.8 |
| 34 | Medical care for adults with IDD is not well coordinated between primary and specialist care and mental health care | 0.504 | 5.7 |
| 57 | Medical care for people with IDD is very heterogenous | 0.428 | 5.8 |
| 67 | There is no agreement about the best approach - whether it is to establish specialized care centers or to support all clinicians to provide good integrated care | 0.291 | 5.2 |
| 68 | Medical care for people with IDD can be provided in the community (at the homes of people with IDD, at their schools) or in medical hospitals | 0.304 | 5.2 |
| | **Cluster 11: Medication use** | 0.4 | 5.1 |
| 17 | Lack of consent for medication is a problem in medical care for people with IDD | 0.462 | 5.0 |
| 18 | People with IDD are not informed properly regarding their diagnosis | 0.346 | 5.5 |
| 28 | Overmedication is a problem for many people with IDD because of the side effects | 0.393 | 5.5 |
| 29 | Overmedication for people with IDD is especially present in long term care facilities | 0.408 | 4.9 |
| 30 | Psychotropic drugs are used too often in the medical care for people with IDD | 0.306 | 4.9 |

| | | | |
|---|---|---|---|
| 71 | It would be good to reduce medicalization of medical care for people with IDD | 0.479 | 4.8 |
| | **Cluster 12: Medical care communication** | 0.54 | 5.6 |
| 21 | Decision support for people with IDD, especially in the medical field, is a problem nowadays | 0.565 | 5.4 |
| 22 | Approaches that aim to increase self-determination of the person with IDD, such as shared decision making and supported decision making are promising practices, but they are only being used by few people in small pilot areas | 0.544 | 5.2 |
| 25 | Treatment is often communicated with whoever is supporting the person with IDD rather than the person him/herself | 0.577 | 5.7 |
| 27 | People with IDD need to have someone to advocate for them in the hospital | 0.471 | 6.2 |
| | **Cluster 13: Attitudinal influences** | 0.69 | 5.0 |
| 3 | There is stigma attached to seeking care from the facilities providing medical care for people with IDD | 1.000 | 4.5 |
| 4 | People with IDD are labelled in society which affects their medical care use | 0.667 | 4.9 |
| 6 | People with IDD are not always protected | 0.801 | 5.5 |
| 26 | People with IDD are always dependent in some way on the people around them and their care system to signal needs and to have access to health care | 0.530 | 5.9 |
| 77 | Restrictive medication rules can hinder people with IDD in travel & activities | 0.447 | 4.1 |

*B=bridging value between 0 and 1

†I=importance rated on a 7-point Likert scale
