# Supplementary material for: Exploring views on medical care for people with intellectual disabilities: an international concept mapping study
Source: Int J Equity Health. 2022 Jul 19;21:99. doi: 10.1186/s12939-022-01700-w (PMC9295354; doi:10.1186/s12939-022-01700-w)

Supplemental Digital Content 3: Point map visualizing the relationship and proximity of statements to one another

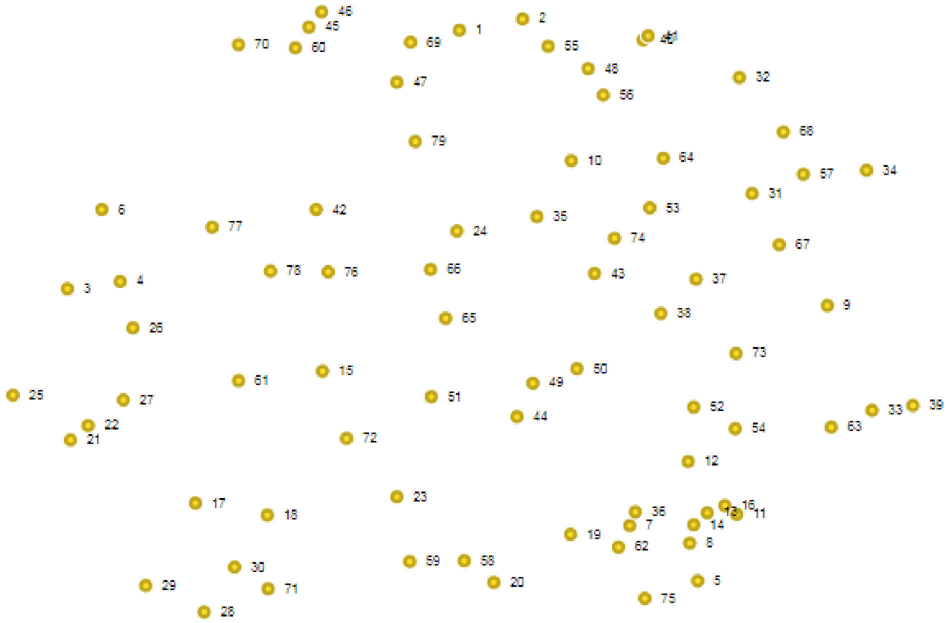

Supplement: Supplementary file 3 — Additional file 3: Supplemental Digital Content 3. Point map visualizing the relationship and proximity of statements to one another. [file 12939_2022_1700_MOESM3_ESM.pdf]
